# Supplementary material for: The genome sequence of the Tortix moth, Archips podanus (Scopoli, 1763)
Source: Wellcome Open Res. 2025 Apr 11;10:189. [Version 1] doi: 10.12688/wellcomeopenres.23921.1 (PMC12361883; doi:10.12688/wellcomeopenres.23921.1)
Supplement: Peer review history

# Reviewer report: Reviewer response for version 1

**DOI:** 10.21956/wellcomeopenres.26390.r127974

**Published:** 2025-08-18

**Authors:** Kay Lucek

**Affiliations:** 1 University of Neuchâtel, Neuchâtel, Switzerland

The authors present the chromosome level genome assembly of a male specimen of the Tortix moth *Archips podanus*. The assembly consists of 30 chromosomes and is not annotated for genes. The assembly is highly complete as revealed by the high BUSCO score but not phased. Sequencing and genome assembly follow the current state of the art and use established methods. The assembly has been generated using the established pipelines of the Darwin Tree of Life Consortium.

 This is a very complete genome note and the genome will be of great value to study this holoarctic species across the Northern Hemisphere, including within its introduced range in North America. The methods sections are very detailed, making the approaches reproducible.

Are sufficient details of methods and materials provided to allow replication by others?

Yes

Is the rationale for creating the dataset(s) clearly described?

Yes

Are the datasets clearly presented in a useable and accessible format?

Yes

Are the protocols appropriate and is the work technically sound?

Yes

Reviewer Expertise:

Speciation genomics

I confirm that I have read this submission and believe that I have an appropriate level of expertise to confirm that it is of an acceptable scientific standard.

# Reviewer report: Reviewer response for version 1

**DOI:** 10.21956/wellcomeopenres.26390.r122696

**Published:** 2025-05-27

**Authors:** Michal Rindoš

**Affiliations:** 1 University of South Bohemia, Ceske Budejovice, Czech Republic

The *Note* contains basic information on the distribution and sexual dimorphism of the species.

 In the abstract I would delete "Tortrix moth" in *Tortix moth; Arthropoda; Insecta; Lepidoptera; Tortricidae*

 and also give examples of the host plants on which the species feeds. Otherwise I think the note is suitable for indexing.

Are sufficient details of methods and materials provided to allow replication by others?

Yes

Is the rationale for creating the dataset(s) clearly described?

Yes

Are the datasets clearly presented in a useable and accessible format?

Yes

Are the protocols appropriate and is the work technically sound?

Yes

Reviewer Expertise:

Biodiversity and systematics of Lepidoptera

I confirm that I have read this submission and believe that I have an appropriate level of expertise to confirm that it is of an acceptable scientific standard.
